# Program evaluation of internet-delivered cognitive behavioral treatments for anxiety and depression in a digital clinic

**DOI:** 10.1016/j.xjmad.2025.100106

**Published:** 2025-01-09

**Authors:** Alexandra L. Silverman, IreLee Ferguson, Jacqueline R. Bullis, Harris Bajwa, Sara Mei, Courtney Beard

**Affiliations:** a Division of Depression and Anxiety, McLean Hospital, Belmont, MA, USA; b Department of Psychiatry, Harvard Medical School, Boston, MA, USA; c Department of Psychology, University of Vermont, Burlington, VT, USA; d Department of Psychology, University of Montana, Missoula, MT, USA; e Department of Psychology, Colorado State University, Fort Collins, CO, USA

**Keywords:** Anxiety, Depression, Digital mental health, CBT, Effectiveness, Engagement

## Abstract

SilverCloud and THIS WAY UP (TWU) are two internet-delivered cognitive-behavioral therapy (iCBT) programs that have demonstrated effectiveness for anxiety and depression, yet little is known about their comparative effectiveness. This non-randomized program evaluation compared client satisfaction, subjective engagement, and treatment outcomes between the SilverCloud and TWU programs. Participants were 195 adult patients (106 using TWU and 89 using SilverCloud) enrolled in a digital CBT clinic who completed assessment measures at pre-treatment, mid-treatment, post-treatment, and one-, two-, three-, and four-month follow-ups. As hypothesized (preregistration: osf.io/x6bmy), patients in both programs reported high client satisfaction, and experienced improvements in depression (*d*s = -0.79 and −0.78), anxiety (*d*s = -0.95 and −0.84), and functional impairment (*d*s = -0.42 and −0.45), from pre- to post-treatment that were maintained at four-month follow-up. However, according to exploratory analyses, the slope of change in treatment outcomes was not significantly different between programs during the treatment and follow-up phases. As hypothesized, patients who used SilverCloud self-reported significantly greater subjective engagement with their iCBT program compared to patients who used TWU (*d*=0.36). However, contrary to hypotheses, client satisfaction was not significantly different between programs. This non-randomized program evaluation offers minimal evidence that one program was better than the other, though findings require replication in a randomized controlled trial and larger sample. Results provide further support for iCBT as a viable option to extend access to high-quality treatment for anxiety and depression.

## Highlights

• First comparison of THISWAYUP vs SilverCloud internet-delivered CBT.
• Improvements in anxiety, depression, and functional impairment within both programs.
• Patients in both programs reported high satisfaction.
• Higher subjective engagement for SilverCloud.
• No significant between-group differences in satisfaction nor treatment outcomes.

## 1 Introduction

Anxiety and depression are highly prevalent and impairing, and represent a significant societal burden [44]. Although effective treatments exist (e.g., cognitive behavioral therapy; CBT), most people with anxiety and depression do not have access to “minimally adequate treatment” [27], [51], in part due to significant barriers that make it difficult to access care (e.g., provider shortages, high therapy costs; [3]). To address this issue, an increasing number of internet-delivered CBT (iCBT) programs for anxiety and depression have been developed in the past two decades, and there is now a substantive body of research demonstrating their positive effects [4]. These programs offer core components of CBT via internet or smartphone, and can be delivered without interaction with a specialist provider, thus increasing convenience and reducing costs for patients in comparison to face-to-face treatments [32]. By circumventing many treatment barriers, iCBT is a more accessible, high-quality treatment option that can be delivered at scale to help fill gaps in care among people who have limited access to high-quality services (e.g., people with low socioeconomic status, or living in remote areas; [32]).

Not surprisingly, healthcare systems around the world have begun to implement iCBT to meet the significant demand for services. For example, the U.K.’s Talking Therapies service offers SilverCloud Health’s iCBT programs for anxiety and/or depression as part of its nationwide stepped-care model [40]. SilverCloud has demonstrated effectiveness in reducing anxiety (*d*s from 0.32 to 0.63), depression (*d*s from 0.50 to 0.55), and functional impairment (*d*s from 0.35 to 0.40) from pre- to post-intervention in comparison to waitlist-control conditions, with symptom levels maintained or improved upon at 6-month follow-up [40], [41].

In Australia, researchers at St. Vincent’s Hospital of Sydney and the University of New South Wales developed THIS WAY UP (TWU; https://thiswayup.org.au/), a series of government-funded iCBT programs for generalized anxiety and/or depression. TWU represents Australia’s largest provider of digital mental health treatment, with more than 217,921 patients enrolling as of 2023 (see https://thiswayup.org.au/research). Effect size estimates for randomized controlled trials (RCTs) comparing TWU to waitlist-control conditions have been large at post-treatment for reductions in anxiety (ranging from 0.78 to 0.85), depression (ranging from 0.49 to 1.00), and functional impairment (ranging from 0.70 to 0.76), and symptom levels have improved or been maintained at 3-month follow-up [29], [49]. More recently, a modified version of TWU was developed for delivery in a large hospital system within the U.S. [42]. Specifically, the language and some minor content were revised to be reflective of American vs. Australian culture, and the program interface was modified to be compliant with the Health Insurance Portability and Accountability Act via confidential access and deidentified self-report data. The modified TWU program demonstrated significantly greater reductions in depression at post-treatment compared to a monitored attentional control condition [42] and was subsequently rolled out as a digital treatment for patients with depression and/or anxiety in the Mass General Brigham (MGB) healthcare system (JRB, personal communication, June 14, 2024).

While TWU and SilverCloud have been scaled up and delivered to thousands of people across healthcare systems globally, these programs have never been directly compared. The present non-randomized program evaluation of a digital CBT clinic evaluated whether there were differences in subjective engagement (i.e., users’ self-reported behavioral, cognitive, and affective engagement; [20]), satisfaction, and treatment outcomes between the TWU and SilverCloud iCBT programs for anxiety and/or depression.

### 1.1 Similarities and differences between iCBT programs

TWU and SilverCloud programs share several important elements, including: (1) online modules that provide psychoeducation on anxiety and/or depression and teach core CBT techniques (e.g., cognitive restructuring, behavioral activation); (2) self-monitoring via mood-monitoring and self-report questionnaires to track symptom change; (3) summaries and homework suggestions at the end of each module; and (4) automated reminders to encourage users to progress. Finally, both programs are typically delivered with low-intensity human support (though this is not required). While the level of support (e.g., when, how often) varies between these programs and across studies, guidance typically consists of brief check-ins with a supportive person (e.g., bachelors-level coach, therapist), completed synchronously via phone or videoconference call, or asynchronously via email or text message, to review the user’s progress, answer questions about the program, and encourage program use [39], [42], [48].

These programs also differ in important ways that may impact users’ subjective engagement, satisfaction, and treatment outcomes. For instance, TWU and SilverCloud vary in terms of how content is displayed and what persuasive technology features they use. TWU modules are presented as illustrated comic-style lessons that follow a fictional character experiencing mental health struggles to learn about how symptoms can be managed using CBT skills [24], while SilverCloud modules contain personal stories from other users and media-rich interactive content (e.g., introductory quizzes, videos, and interactive activities; [39]). Further, the SilverCloud platform can be used to send and receive messages from a coach, thus allowing additional guidance to be offered asynchronously to users based on their needs [39], which is not possible on the TWU platform. Prior reviews have found that incorporating interactive features, personal stories from successful users, and text messages into digital mental health interventions can enhance user engagement and experiences [23], [6]. Thus, given Silvercloud’s greater use of features that facilitate engagement, it is possible that SilverCloud (vs. TWU) users may perceive their user experience as more engaging and satisfying.

TWU and SilverCloud also differ in their structure and delivery. While TWU can be completed via web-based platform only, SilverCloud can be completed via web-based platform and/or Smartphone application, which may make it easier for people to use SilverCloud when and where it is most convenient. Additionally, TWU programs were designed to more closely mimic face-to-face CBT, and recommend completing one treatment module per week in one 45–60 minute sitting, whereas SilverCloud recommends more bite-sized interactions with their programs (e.g., 10–15 minutes two to three times weekly). Additionally, TWU follows a tunneled (i.e., sequential) structure: modules follow a pre-determined order and progressively build on the content of previous modules; and users must wait at least five days before completing the next module in the sequence, during which time they are encouraged to complete homework exercises to practice CBT skills and consolidate learning. In contrast, SilverCloud uses a free-roam structure that allows users to complete modules in any order without following a pre-determined schedule. Thus, while SilverCloud users are encouraged to complete at least one module per week, they can complete multiple modules simultaneously, and in any order, without waiting to start additional modules.

Importantly, these structural differences may influence how users engage with these programs, as well as their level of satisfaction with their experience. iCBT programs with free-roam (vs. tunneled) structures offer users more control and autonomy over what content they view and in what order, which may increase their subjective engagement and satisfaction [36]. Indeed, prior qualitative studies of SilverCloud and other mental health apps have found that many users appreciate having control over their pace of using the program, and the ability to complete modules in any order [1], [19], [35]. Furthermore, SilverCloud’s free-roam structure may enable people to use the program in ways that best fit their needs, which may result in greater satisfaction, subjective engagement, and treatment outcomes [6]. Alternatively, people may find that the increased autonomy offered by SilverCloud results in them completing fewer modules, feeling overwhelmed, or skipping potentially important material. In support of this hypothesis, qualitative research has found that SilverCloud’s lack of structure has made it easier for some users to put off and forget about the program, and can lead to confusion about what should be worked on from day-to-day [19], [35]. In contrast to SilverCloud’s free-roam structure, TWU’s tunneled structure may help ensure that users complete treatment components that may not otherwise be viewed. Further, having to wait five days between TWU modules creates a designated period for CBT skills practice and homework completion to encourage the consolidation of learning and integration of skills into daily life, which (if done accordingly) may enhance treatment outcomes.

Despite robust empirical support for the effectiveness of iCBT, including meta-analyses indicating that guided iCBT can achieve comparable results to face-to-face CBT for anxiety and depression [2], [9], engagement is often poor [14], [30]. Therefore, further research into the relationship between program structure, subjective engagement, satisfaction, and treatment outcomes may yield important insights into how the potential of iCBT programs can be more fully realized. To date, no study has directly compared outcomes between tunneled and free-roam iCBT programs, and existing evidence for the manner in which digital interventions should be structured is inconclusive [34], [37]. This study takes a preliminary step to fill this gap by evaluating differences between tunneled (TWU) and free-roam (SilverCloud) iCBT offered in a digital clinic.

### 1.2 Study overview and hypotheses

In the present non-randomized study, we conducted a program evaluation of a digital clinic delivering guided iCBT to adults with mild-to-moderate anxiety and/or depression symptoms. We evaluated subjective engagement, satisfaction, and changes in anxiety, depression, and functional impairment over time within and between SilverCloud and MGB’s adaptation of TWU (herein referred to as TWU).

Hypotheses were pre-registered at Open Science Framework (osf.io/x6bmy). For analyses of client satisfaction and subjective engagement, we hypothesized that: (a) both iCBT programs would meet benchmarks for satisfaction (i.e., total scores on the Client Satisfaction Questionnaire-8 of greater than or equal to 24; [33]); and (b) patients using SilverCloud (vs. TWU) would report greater satisfaction and subjective engagement with their respective program, given that SilverCloud (vs. TWU) contains more features that enhance user experience and engagement. For analyses of treatment effectiveness, we hypothesized that both iCBT programs would demonstrate significant reductions in anxiety, depression, and functional impairment over time. Finally, we did not have specific hypotheses for analyses comparing treatment outcomes between SilverCloud and TWU, given that no prior studies have directly compared these iCBT programs and both programs have previously demonstrated effectiveness when compared to waitlist-control conditions. Thus, these analyses were considered exploratory.

## 2 Method

### 2.1 Participants

**Fig. 1:**
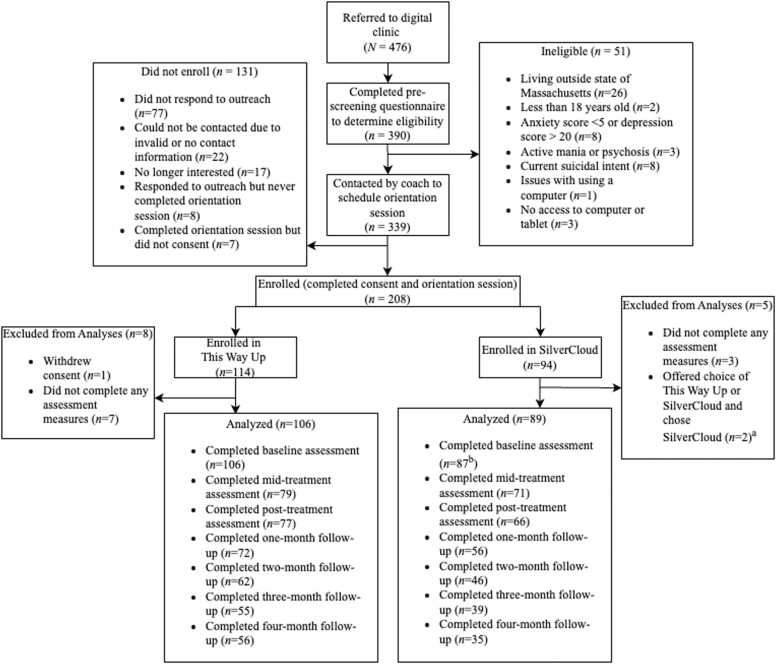
CONSORT flow diagram. Note. CBT = cognitive-behavioral therapy; ^a^Between January 10, 2021 and January 24, 2021, patients were offered their choice of internet-delivered CBT program. Two patients who chose to enroll in SiverCloud during this period were excluded from analyses, given concerns that choosing one’s preferred program might impact outcomes of interest [47]. ^b^Two patients did not complete pre-treatment measures of depression, anxiety, and functional impairment.

Participants were English-speaking adults (18 years or older) residing in Massachusetts who enrolled in a digital CBT clinic. The digital CBT clinic used several screeners to guide eligibility decisions. With few exceptions, people were enrolled if they had: (a) access to reliable internet and a computer or Smartphone to complete the iCBT program; (b) a total score ≥ 5 and < 20 on the Patient Health Questionnaire-9 (PHQ-9; [22]), indicating mild-to-moderate depression symptoms; and/or a total score ≥ 5 on the Generalized Anxiety Disorder-7 (GAD-7; [46]), indicating at least mild anxiety symptoms. In general, people were referred to more appropriate services if they reported: (a) current suicidal intent or behaviors; (b) active mania or psychosis, or (c) significant alcohol and/or substance use that would make it difficult to participate in and benefit from the program. Analyses were conducted among the intent-to-treat sample of 195 patients who enrolled and completed their initial orientation session (TWU: *n* = 106; SilverCloud: *n* = 89). Patients in the sample were middle-aged on average (*M*=51.63, *SD*=16.48), and identified predominantly as women (81.0 %), non-Hispanic White (86.2 %), and college educated (88.2 %). See Fig. 1 for the CONSORT Flow Diagram and Table 1 for sample characteristics.

**Table 1:** Demographic and clinical characteristics.

Characteristic	This Way Up (*n* = 106)	SilverCloud (*n* = 89)	Difference Statistics and/or *p* values^a^
Age: *M* (*SD*)	52.27 (16.05)	50.86 (17.06)	*W* = 4659, *p* = .706
Gender: *n* (%)			*p* = .100
Woman	87 (82.1)	71 (79.8)	
Man	18 (17.0)	14 (15.4)	
Unknown or Prefer not to answer	1 (0.9)	4 (4.5)	
Sex assigned at birth: *n* (%)			*p* = .100
Female	86 (83.0)	73 (82.0)	
Male	18 (17.0)	14 (15.7)	
Unknown or Prefer not to answer	0 (0.0)	2 (2.3)	
Racial-Ethnic Identity: *n* (%)			*p* = 1.00
Non-Latinx White	92 (86.8)	76 (85.4)	
Latinx-White	2 (1.9)	1 (1.1)	
Non-Latinx Black	1 (0.9)	2 (2.3)	
Latinx-Black	1 (0.9)	1 (1.1)	
Non-Latinx Asian	3 (2.9)	1 (1.1)	
Non-Latinx Multiracial	2 (1.9)	3 (3.4)	
Unknown or Prefer not to answer	5 (4.7)	5 (5.6)	
Education: *n* (%)			*p* = .355
Less than four-year college graduate	9 (8.5)	12 (13.5)	
Four-year college graduate	41 (38.7)	27 (30.3)	
Post-college education	55 (51.9)	52 (51.1)	
Unknown or Prefer not to answer	1 (0.9)	1 (1.1)	
Sexual Orientation: *n* (%)			
Heterosexual	96 (90.5)	77 (86.5)	*p* = .572
Gay/lesbian	2 (1.9)	0 (0.0)	
Bisexual	2 (1.9)	5 (5.6)	
Another sexual orientation	2 (1.9)	2 (2.3)	
Unknown or Prefer not to answer	4 (3.8)	5 (5.6)	
Employment Status: *n* (%)			*p* = .656
Full time	52 (49.0)	40 (44.9)	
Part time	15 (14.2)	14 (15.7)	
Unemployed	11 (10.3)	7 (7.9)	
Student	2 (1.9)	6 (6.7)	
Retired	19 (17.9)	17 (19.1)	
Homemaker	4 (3.8)	3 (3.4)	
Unknown or Prefer not to answer	3 (2.9)	2 (2.3)	
Income: *n* (%)			
Less than $40,000	14 (13.2)	15 (16.9)	*W* = 4680.5, *p* = .673
$40,000 – $60,000	8 (7.6)	10 (11.2)	
$60,000 – $80,000	11 (10.4)	8 (9.0)	
$80,000 – $100,000	9 (8.5)	4 (4.5)	
Greater than $100,000	38 (35.8)	29 (32.6)	
Unknown or Prefer not to answer	26 (24.5)	23 (25.8)	
Baseline Clinical Characteristics			
Depression: *M* (*SD*)	8.46 (4.23)	8.65 (4.70)±	*W* = 4564, *p* = .904
Anxiety: *M* (*SD*)	9.83 (4.61)	8.81 (5.16)^b^	*W* = 5248 *p* = .098
Functional Impairment: *M* (*SD*)	16.08 (9.32)	14.32 (9.09)^b^	*W* = 5201.5, *p* = .126

a Fisher's exact tests were used to test associations between categorical variables, which do not produce a test statistic.

± Two patients did not answer all depression, anxiety, and functional impairment items at baseline.

### 2.2 Digital CBT Clinic

The digital CBT clinic began in February 2021 through a partnership with the Combined Jewish Philanthropies (CJP), Jewish Family and Children’s Services (JF&CS), and McLean Hospital and is currently still active. CJP and JF&CS are philanthropic organizations that provide community resources, including mental health support, focused on serving, but are not exclusive to, the Jewish community.

#### 2.2.1 iCBT programs

Since 2021, different iCBT programs were offered by the digital clinic. From February 4, 2021, through January 9, 2022, all patients for the present study were enrolled in TWU. In early 2022, MGB transitioned to a new digital CBT program, SilverCloud. During this transition period (January 10, 2021 through January 24, 2021), patients were offered their choice of TWU or SilverCloud. Two patients who chose to enroll in SiverCloud during this period were excluded from analyses, given concerns that choosing one’s preferred iCBT program might impact relevant outcomes [47].^1^ All patients after January 24, 2022 through the endpoint for data collection (July 31, 2024), were enrolled in SilverCloud. See Table S1 for a summary of modules/CBT skills covered by each program.

#### 2.2.2 TWU

TWU delivers web-based programs that have been described in detail elsewhere [42]. One of three programs was offered to patients based on their PHQ-9 and GAD-7 scores at pre-screening: a depression-specific program was offered to patients with a PHQ-9 score ≥ 5 and GAD-7 score < 5 (*n* = 1); an anxiety-specific program was offered to patients with a GAD-7 score ≥ 5 and PHQ-9 score < 5 (*n* = 12); and a mixed anxiety and depression program was offered to patients with PHQ-9 and GAD-7 scores ≥ 5 (*n* = 93). All three programs consisted of six 45–60-minute modules completed in sequential order over eight weeks, with a required five-day waiting period between modules. A summary sheet and homework exercises were provided at the end of each module. In this study, a modified U.S. version of TWU was used [42].

#### 2.2.3 SilverCloud health

SilverCloud Health delivers CBT-based programs that can be accessed via web-based program or smartphone application, and are described in detail elsewhere [39]. One of three programs was offered to patients based on their PHQ-9 and GAD-7 scores at pre-screening: a depression-specific program was offered to patients with a PHQ-9 score ≥ 5 and GAD-7 score < 5 (*n* = 1); an anxiety-specific program was offered to patients with a GAD-7 score ≥ 5 and PHQ-9 score < 5 (*n* = 5); and a mixed program for depression and anxiety was offered to patients with PHQ-9 and GAD-7 scores ≥ 5 (*n* = 83). All three programs consisted of eight hour-long core modules covering fundamental CBT principles. Additional modules could also be unlocked by a coach that covered more specialized topics (e.g., anger management, grief). Given that program content is delivered non-sequentially, SilverCloud does not capture information on users’ completion of each module. Adherence is instead tracked via alternative metrics, including number of logins, time spent using the program, and unique modules viewed. (Note that if a module was viewed, this means that least one page of the module was viewed and does not indicate whether the module was completed).

#### 2.2.4 Coaching

The coaching protocol was consistent with coaching models described in prior iCBT studies (e.g., [39], [42]) and provided by three bachelors-level coaches who were trained and supervised by a licensed clinical psychologist (JRB). All coaching calls were delivered via phone or videoconference call based on the patient’s preference. During the initial call, informed consent was obtained, and patients completed an orientation session. Following the orientation, coaching check-in calls were completed once per week for eight weeks. Check-in calls lasted approximately 15 minutes and were used to provide technical support, answer questions about how and when to use CBT skills and offer support in goal setting (see S1.1 for additional details on training and supervision of coaches, and coaching procedures).

### 2.3 Measures

#### 2.3.1 Satisfaction

Patients completed the Client Satisfaction Questionnaire-8 (CSQ-8; [33]) at post-treatment. The CSQ-8 is an eight-item self-report measure of satisfaction with the “services received” that uses a four-point scale from one to four, with different anchors depending on each question. A total score was calculated, with higher scores indicating greater treatment satisfaction. Internal consistency for the TWU and SilverCloud groups was excellent (αs and 95 % CI=.90, [.87,.93] and.90[.87,.93]).

#### 2.3.2 Subjective engagement

Patients completed the Twente Engagement with eHealth Technologies Scale (TWEETS; [20]) at post-treatment. The TWEETS is a nine-item self-report measure that assesses cognitive, affective, and behavioral engagement with eHealth technologies using a five-point scale from 0 (*strongly disagree*) to 4 (*strongly agree*). The TWEETS allows for adaptation to the studied technology by adding the technology, goal, and behavior related to the goal to the items. This was implemented for this study as “the CBT program,” “to develop healthier mental habits”, and “get more insight into my depression/anxiety symptoms.” Following Kelders et al. [20], the mean across all nine items was calculated, with higher scores indicating greater subjective engagement. Internal consistency was excellent for the TWU group, and good for the SilverCloud group (αs and 95 % CIs=.92[.90,.94] and.88[.83,.91]).

#### 2.3.3 Treatment effectiveness

##### 2.3.3.1 Depression symptoms

The PHQ-9 [22] is a 9-item self-report questionnaire that assesses depression symptoms on a 4-point scale from 0 (*not at all*) to 3 (*nearly every day*). Patients completed the PHQ-9 at screening for eligibility, pre-treatment (baseline), mid-treatment (i.e., after one month), post-treatment (i.e., after two months), one-month follow-up, two-month follow-up, three-month follow-up, and four-month follow-up. A total score was calculated, with higher scores indicating greater depression symptom severity. Internal consistency for the TWU and SilverCloud groups was acceptable at pre-treatment (αs and 95 % CIs=.73[.65,.80] and.78[.71,.84]).

##### 2.3.3.2 Anxiety symptoms

The GAD-7 [46] is a seven-item self-report questionnaire that assesses anxiety symptoms using a 4-point scale from 0 (*not at all*) to 3 (*nearly every* day). Patients completed the GAD-7 at screening for eligibility, pre-treatment, mid-treatment, post-treatment, one-month follow-up, two-month follow-up, three-month follow-up, and four-month follow-up. A total score was calculated, with higher scores indicating greater anxiety symptom severity. Internal consistency for the TWU and SilverCloud groups was good at pre-treatment (αs and 95 % CIs=.85[.80,.89] and.88[.84,.92]).

##### 2.3.3.3 Functional impairment

The Work and Social Adjustment Scale (WSAS; [28]) is a five-item self-report questionnaire that assesses a person’s impairment and experiential impact across five domains (work, social life, home life, private life, and close relations) using a nine-point scale from 0 (*no impairment at all*) to 8 (*very severe impairment*). Patients completed the WSAS at pre-treatment, post-treatment, one-month follow-up, two-month follow-up, three-month follow-up, and four-month follow-up. A total score was calculated, with higher scores indicating greater functional impairment. Internal consistency for the TWU and SilverCloud groups was good at pre-treatment (αs and 95 % CIs=.87[.83,.91] and.89[.86,.93]).

### 2.4 Procedure

This study was approved by the MGB Institutional Review Board. Potential patients were referred by JF&CS’s free, confidential referral service, or referred themselves using JF&CS’s or CJP’s websites. Referred patients completed a brief pre-screening questionnaire via Research Electronic Data Capture (REDCap), a secure, web-based application designed to capture data for research studies [17], to assess their initial eligibility. If there were any questions about eligibility (e.g., symptom severity, possible recent history of mania), coaches discussed concerns with the supervising psychologist to make a final determination of eligibility. Eligible patients were then asked to schedule an initial coaching call, in which they completed the informed consent process, and (if consented) a 20- to 40-minute orientation. During the orientation, patients created an iCBT program account, logged into the program and received guidance on how to use it, and scheduled their first 15-minute coaching check-in call. TWU patients were encouraged to complete one module per week (which required approximately 45–60 minutes), whereas SilverCloud patients were encouraged to use the program in 10- to 15-minute increments two to three times per week (for a total time of 20–45 minutes).

Assessment measures were completed via REDCap surveys sent to patients via email. Patients completed assessment measures at baseline (i.e., following orientation, before starting any iCBT modules), half-way through treatment, at post-treatment, and at one-month, two-month, three-month, and fourth-month follow-ups.

### 2.5 Data analysis

Analyses were conducted in R (Version 4.2.0; [38]). Power analyses were conducted prior to data analysis to determine whether we would be adequately powered to test hypotheses assuming 106 TWU patients and 91 SilverCloud patients. For analyses of satisfaction and subjective engagement, G*Power (Version 3.1; [10]) was used. For analyses of treatment effectiveness, power analyses by simulation were conducted using the mlmpower R package [21]. Power analyses indicated that the study had at least 80 % power to detect anticipated effect sizes (i.e., medium for satisfaction and subjective engagement, small for treatment effectiveness) for all outcomes (see S1.2 and Table S2 for detailed results).

#### 2.5.1 Baseline group differences

Given that patients were not randomized to iCBT program, we examined whether the TWU and SilverCloud groups differed based on demographic and baseline clinical characteristics. We used Fisher’s exact tests for categorical variables, and Wilcox rank-sum tests for continuous variables. We planned a priori to include any variables that significantly differed between groups as covariates in all subsequent between-group analyses.

#### 2.5.2 Missing data

The data presented a missing data pattern at the scale level due to attrition (i.e., patients withdrawing from treatment or noncompliance with follow-up surveys). The proportions of scale-level missing data across the five outcomes ranged from 29.7 % to 35.4 % (see Table S3 for the number of missing observations for each outcome). To identify measured variables other than time that may relate to this pattern of missing data, we evaluated whether any demographic variables were related to missingness (see S1.3 for results). Education was the only significant predictor. As such, the missing completely at random assumption was not met, and multiple imputation was used to account for missing data. Education was included as an auxiliary variable in the multiple imputation model to correct for any systematic bias resulting from this variables’ relationship with missingness (see S1.4 for description of the multiple imputation model).

#### 2.5.3 Client satisfaction and subjective engagement

To examine the effect of iCBT program on client satisfaction and subjective engagement, we tested pooled independent samples t-tests with the imputed data, with program (TWU vs. SilverCloud) entered as the predictor variable, and client satisfaction (CSQ-8 total scores) and subjective engagement (TWEETS mean scores) entered into separate models as the dependent variable.

#### 2.5.4 Treatment effectiveness

We used hierarchical linear models to evaluate treatment outcomes within and between iCBT programs. Anxiety, depression, and functional impairment served as individual outcomes. We assumed piecewise linear trajectories (one during the treatment period: time_TR_; one during the follow-up period: time_FU_) because we expected that the rate of change would differ between the treatment and follow-up periods, such that we would observe greater changes in outcomes during versus after treatment. A dummy-coded covariate was included in all models to control for whether the program was mixed anxiety and depression vs. anxiety- or depression-specific (collapsing across anxiety- and depression-specific programs, given the small sample size in each group).

##### 2.5.4.1 Between-group effects

In each model, we simultaneously entered fixed effects for group, time_TR_, time_FU_, and the group × time_TR_ and group × time_FU_ interactions and random effects for intercept, time_TR_, and time_FU_. For the functional impairment model, we did not include a random effect for time_TR_ because the WSAS was only measured at two timepoints during the treatment period. Group (TWU vs. SilverCloud) was dummy-coded, with TWU serving as the reference group. For significant interactions, we planned to assess the simple effects of time at the two group levels, using separate models with fixed effects of time_TR_ and time_FU_, and random effects for intercept, time_TR_, and time_FU_.

##### 2.5.4.2 Within-group effect of time

In a separate dataset, we assessed the simple effects of time_TR_ and time_FU_ in separate models for each iCBT program to understand the overall change over time in outcomes, regardless of the interactions’ significance.

##### 2.5.4.3 Effect sizes

The size of group differences for model-estimated means at post-treatment and four-month follow-up assessments between the TWU and SilverCloud programs was computed as growth-modeling analysis *d* (GMA *d*; [11]), which has the same metric as Cohen’s *d.* We computed time-specific GMA *d* (adapted from [12]) at post-treatment and four-month follow-up to describe group differences at the end of treatment and at the end of the follow-up period, respectively. We also computed within-group GMA *d* at post-treatment and four-month follow-up to describe changes in outcomes within each iCBT program.

## 3 Results

### 3.1 Baseline group differences

There were no significant differences in any demographic or baseline clinical characteristics between programs, all *p*s > .05 (see Table 1).

### 3.2 Adherence

Rates of each module completed (TWU) or viewed (SilverCloud), and adherence to each weekly coaching check-in are presented in Table 2. On average, TWU patients completed 4.97 modules (*SD*=1.93, range=0–6), which is equivalent to an average completion of 82.9 % of all program content. SilverCloud patients viewed an average of 6.26 core modules (*SD*=2.47, range=0–8), had an average 41.99 logins (*SD*=48.78) and spent 9 h 52 min on the platform (*SD*=596 min). In terms of coaching adherence, TWU patients completed 6.04 check-ins (*SD*=2.45, range=0–8), and SilverCloud patients completed 6.07 check-ins (*SD*=2.44, range=0–8).

**Table 2:** Rates of iCBT modules completed/viewed and coaching check-ins completed.

	THIS WAY UP (*n* = 106)		SilverCloud (*n* = 89)	
Frequency	Modules Completed *n* (%)	Coaching Sessions Completed *n* (%)	Modules Viewed *n* (%)	Coaching Sessions Completed *n* (%)
0	6 (5.7)	8 (7.6)	2 (2.2)	5 (5.6)
1	7 (6.6)	4 (3.8)	5 (5.6)	5 (5.6)
2	4 (3.8)	1 (0.9)	4 (4.5)	1 (1.1)
3	4(3.8)	3 (2.8)	5 (5.6)	2 (2.3)
4	2 (1.9)	4 (3.8)	5 (5.6)	5 (5.6)
5	6 (5.7)	7 (6.6)	3 (3.4)	8 (9.0)
6	77 (72.6)	17 (16.0)	4 (4.5)	8 (9.0)
7	--	24 (22.6)	13 (14.6)	21 (23.6)
8	--	38 (35.9)	45 (50.6)	30 (33.7)

*Note*. iCBT = internet-delivered cognitive-behavioral therapy; THIS WAY UP had six modules, while SilverCloud had eight core modules.

### 3.3 Client satisfaction and subjective engagement

The a priori benchmark for client satisfaction (total score of 24 or greater on the CSQ-8) was achieved in both the TWU (*M*=25.81, *SD*=4.13) and SilverCloud programs (*M*=26.93, *SD*=4.18). Contrary to hypotheses, client satisfaction did not differ significantly between programs, *t*(121) = 1.82, *p* = .071, *d*= 0.27. As hypothesized, SilverCloud patients reported significantly greater subjective engagement (*M*=2.79, *SD*=0.59) compared to TWU patients (*M*=2.55, *SD*=0.75), *t*(118.83) = 2.23, *p* = .028, *d*= 0.36.

### 3.4 Treatment effectiveness

#### 3.4.1 Within-group effects

See Table 3 for results for models testing within-group effects of change in outcomes over the treatment and follow-up phases for both groups. As hypothesized, both groups experienced statistically significant reductions from pre- to post-treatment for all outcomes (*p*s < .001), with medium effect sizes for depression (*d*s = -0.79 and −0.78), large effect sizes for anxiety (*d*s = -0.95 and −0.84), and small effect sizes for functional impairment (*d*s = -0.42 and −0.45). Both groups showed no significant change from post-treatment to four-month follow-up in depression, anxiety, and functional impairment. However, within-group effect sizes from pre-treatment to four-month follow-up remained negative, with medium effect sizes for depression (*d*s = -0.59 and −0.73), anxiety (*d*s = -0.76 and −0.79), and functional impairment (*d*s = -0.54 and −0.57), suggesting that as hypothesized, patients improved during the treatment phase and maintained their gains during the follow-up period.

**Table 3:** Linear spline multilevel models for fixed group × time interaction effects and time effects in each group.

Outcome	Phase	Effect	B (SE)	95 % CI		*p*	GMA *d*	
Depression	TR	Group × Time	−0.29 (0.31)	[−0.90, 0.32]	.353			−0.13
		Time_TWU_	−1.66 (0.41)	[−2.10, −1.22]	< .001			−0.79
		Time_SilverCloud_	−1.95 (0.25)	[−2.45, −1.45]	< .001			−0.78
	FU	Group × Time	−0.13 (0.21)	[−0.56, 0.29]	.533			−0.24
		Time_TWU_	0.20 (0.17)	[−0.14, 0.55]	.247			−0.59
		Time_SilverCloud_	0.07 (0.16)	[−0.25, 0.39]	.658			−0.73
Anxiety	TR	Group × Time	0.02 (0.30)	[−0.57, 0.61]	.951			0.01
		Time_TWU_	−2.18 (0.23)	[−2.64, −1.72]	< .001			−0.95
		Time_SilverCloud_	−2.16 (0.16)	[−2.72, −1.61]	< .001			−0.84
	FU	Group × Time	−0.15 (0.19)	[−0.52, 0.22]	.434			−0.11
		Time_TWU_	0.21 (0.12)	[−0.04, 0.46]	.095			−0.76
		Time_SilverCloud_	0.07 (0.16)	[−0.26, 0.39]	.694			−0.79
Functional Impairment	TR	Group × Time	−0.13 (0.50)	[−1.11, 0.85]	.799			−0.03
		Time_TWU_	−1.97 (0.33)	[−2.62, −1.31]	< .001			−0.42
		Time_SilverCloud_	−2.10 (0.35)	[−2.79, −1.40]	< .001			−0.45
	FU	Group × Time	−0.11 (0.35)	[−0.68, 0.66]	.983			−0.03
		Time_TWU_	−0.27 (0.27)	[−0.81, 0.28]	.332			−0.54
		Time_SilverCloud_	−0.27 (0.25)	[−0.77, 0.23]	.281			−0.57

*Not*e. Separate models were fit for the analyses of between-group interactions and within-group effects of time. GMA = Growth-modeling analysis; TWU = THIS WAY UP; TR = treatment; FU = follow-up.

#### 3.4.2 Between-group effects

**Fig. 2:**
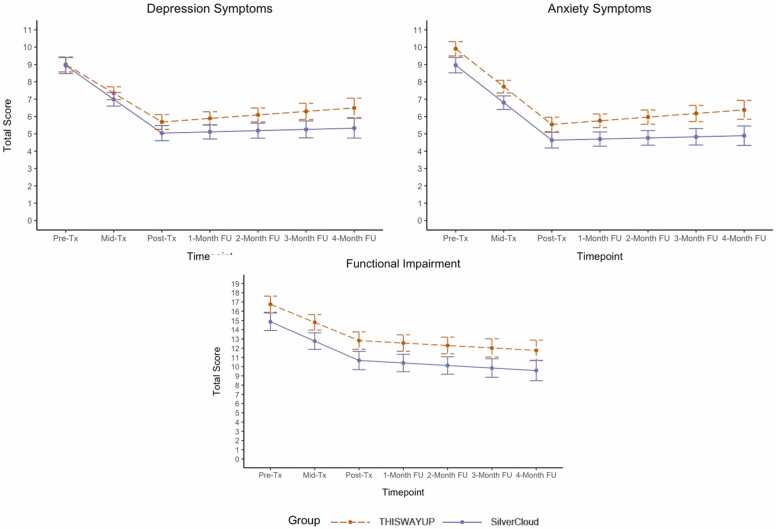
Linear spline estimated means for treatment outcomes over time by group. Note. Tx = Treatment; FU = Follow-up. Means ( ± 1 SE) estimated from the linear spline multilevel model of between-group effects with THIS WAY UP as the reference group are shown. Estimates were computed from each imputed dataset and then pooled following Rubin’s rules using the testConstraints function of the mitml package [16] in R.

Results for exploratory analyses testing group × time interaction effects during the treatment and follow-up phases are reported in Table 3 and graphically displayed in Fig. 2. No significant slope differences emerged between the TWU and SilverCloud groups from pre- to post-treatment for all three outcomes (depression, anxiety, and functional impairment), and effect sizes for the mean difference between groups from pre- to post-treatment were negligible (*d*s ranging from −0.13 to −0.01). There were also no significant slope differences between groups from post-treatment to four-month follow-up. Effect sizes for the mean difference between groups from pre-treatment to four-month follow-up were negligible for anxiety (*d*=-0.11) and functional impairment (*d*=-0.03), and small for depression (*d*=-0.24). Findings remained the same after controlling for baseline symptom levels (see S2.1 and Table S4 for results of sensitivity analyses).

## 4 Discussion

This non-randomized program evaluation is the first study to compare satisfaction, subjective engagement, and treatment outcomes between the TWU and SilverCloud iCBT programs for anxiety and/or depression. As hypothesized, patients in both programs met benchmarks for client satisfaction and experienced significant improvements in anxiety, depression, and functional impairment during the treatment phase that were maintained at four-month follow-up. Further, in line with hypotheses, patients who used SilverCloud (vs. TWU) reported significantly greater subjective engagement with their respective program. However, contrary to hypotheses, client satisfaction did not significantly differ between programs, and exploratory analyses revealed no significant between-group differences in treatment effectiveness during the treatment or follow-up phases.

Regarding effectiveness, symptoms of depression, anxiety and functional impairment decreased over time within both programs, with medium, large, and small effect sizes, respectively, from pre- to post-treatment, and medium effect sizes across all three outcomes from pre-treatment to four-month follow-up. These effect sizes are consistent with within-group effects observed in prior trials of TWU and SilverCloud conducted in clinical practice settings (e.g., [31], [35]), as well as in a recent comparative effectiveness RCT evaluating SilverCloud against mindfulness-based and enhanced personalized feedback digital mental health interventions [18]. This evidence further supports the real-world value of integrating iCBT into U.S. healthcare systems [26].

In terms of adherence, 80.2 % of TWU patients completed four or more iCBT modules and were therefore exposed to most CBT content. This adherence rate is higher than those from other real-world TWU evaluations (ranging from 46.4 % to 70.0 %; [25], [30]). It was not possible to compute module completion for SilverCloud patients. However, this may not be the most informative adherence metric for a free-roam iCBT program like SilverCloud that encourages bite-size interactions with the program. To this end, one study found that completion of 50 % of SilverCloud program content was associated with a higher probability of reliable change [8]. As such, the field’s understanding of effective adherence is shifting away from a “the more usage, the better” stance to determining how much use is enough to produce clinical benefits [45]. Nonetheless, the available adherence metrics for SilverCloud patients were encouraging (average of 41.99 logins and 592 min spent on the platform), and compared favorably to prior studies from practice settings (e.g., 14.3–17.4 logins and 295.7–553.2 min spent on the platform; [35], [39], [40]).

As hypothesized, SilverCloud (vs. TWU) patients reported greater subjective engagement with their respective program. Yet, despite this, client satisfaction and treatment effectiveness did not differ significantly between programs. This pattern of findings is surprising given that subjective engagement has been proposed as a target mechanism through with digital mental health interventions achieve clinical outcomes [15], [36]. It is worth noting that while client satisfaction did not significantly differ between programs, there was a small effect size (*d*=0.27) in the expected direction such that SilverCloud (vs. TWU) patients reported greater satisfaction with their respective program. Given that we outlined a priori the aim for this analysis to be powered to detect a moderate effect size (*d*=0.50) between programs, we may not have been adequately powered to detect small effects. It is thus possible that the effect size observed in this study would be statistically significant in a larger sample, and replication of this analysis in a sample that is adequately powered to detect small effect sizes is warranted.

If the present pattern of findings is replicated, one possible explanation for the discrepant findings across outcomes is that coaching (a *non-specific* treatment component that was consistent across programs) may have had a more substantive impact on treatment effectiveness than the treatment components that differed between programs (e.g., structure, interactive features). It is not possible to disentangle the effects of coaching from the effects of the iCBT programs in this study, and findings from prior research examining the impact of coaching on treatment effectiveness are relatively mixed [5]. Dismantling studies (e.g., [13]) are needed to clarify whether outcomes change when coaching is added/removed, and the Multiphase Optimization Strategy (MOST; [7]) can be used to determine iCBT components (e.g., coaching, specific program features) that optimize engagement and outcomes.

Although the TWEETS is recommended for use as a single scale [20], post-hoc analyses of the behavioral, cognitive, and affective subscales were used to shed light on the present study findings (see S2.2 for results). The TWEETS measures behavioral engagement (i.e., using the technology is part of one’s daily routine, takes little effort, and can be done as often as needed to achieve one’s goals), cognitive engagement (i.e., feeling motivated and supported by the technology to reach one’s goals), and affective engagement (i.e., sense of enjoyment and identity fit with the technology; [20]). Post-hoc analyses indicated that SilverCloud (vs. TWU) patients reported significantly greater behavioral and cognitive engagement, whereas affective engagement did not significantly differ between groups. Further, across both programs, client satisfaction correlated the most highly with affective engagement, followed by cognitive engagement, and last with behavioral engagement. Taken together, these findings suggest that between-group differences in subjective engagement are explained more by differences in behavioral and cognitive engagement than by differences in affective engagement (which is most related to client satisfaction).

SilverCloud (vs. TWU) patients may have reported greater subjective behavioral and cognitive engagement for several reasons. SilverCloud’s use of a free-roam (vs. tunneled) structure and ability to be completed via computer or Smartphone (vs. computer only) might lend the program greater behavioral engagement, while the program’s use of interactive features may enhance cognitive engagement [36]. Given that this was a nonrandomized study without a control condition, and since the TWU and SilverCloud programs differed in many ways that could impact subjective engagement, we cannot draw conclusions about what specific aspects of the iCBT programs influenced subjective engagement. RCTs that use an experimental therapeutics framework to evaluate subjective engagement as a treatment target are needed to clarify the relationship between subjective engagement and outcomes [15], and to shed light on how iCBT programs can be designed to maximize their effectiveness. Further, research is needed to examine which iCBT elements best engage and optimize outcomes for people with specific attributes (e.g., motivation for treatment, lifestyle) to clarify what characteristics make someone a better candidate for SilverCloud vs. TWU and help inform treatment recommendations.

### 4.1 Limitations

This study was limited by the lack of randomization to condition. While there were no significant differences in demographic and baseline clinical characteristics between groups, it is not possible to rule out the role of other variables that may have confounded results. For instance, there may have been cohort effects, given that TWU patients completed treatment at an earlier stage in the COVID-19 pandemic compared to SilverCloud patients [25]. Further, most patients received iCBT for mixed anxiety and depression symptoms rather than a depression- or anxiety-focused program. While analyses included a covariate to account for iCBT program type, results may not generalize to disorder-specific iCBT programs or populations. Additionally, without a control condition, we cannot rule out the possibility that symptom reductions were accounted for by other factors (e.g., spontaneous recovery). Further, while a priori power analyses indicated that the study was adequately powered to detect the anticipated (medium) effect size for the analysis of client satisfaction, the sample size may not have been powered to detect significant effects for smaller effect size differences between programs. Moreover, the sample predominantly consisted of non-Hispanic White, highly educated women. This is primarily due to the demographic characteristics of people served by the digital clinic’s community partners and also reflects the typical user of digital mental health interventions [50]. Efforts are needed to evaluate the effectiveness and acceptability of these programs in diverse samples, especially given prior research indicating that rates of iCBT use are consistently lower among racial and ethnic minorities relative to what would be expected based on race/ethnicity population estimates [43]. Finally, each iCBT program captured different adherence metrics, which prevented a direct comparison of adherence between programs.

## 5 Conclusions

To our knowledge, this is the first attempt at comparing outcomes between TWU and SilverCloud, two well-established and commonly implemented iCBT programs for treatment of anxiety and depression. While subjective engagement was higher among SilverCloud (vs. TWU) patients, patients in both programs reported high satisfaction and improvements in depression, anxiety and functional impairment at post-treatment and four-month follow-up. If replicated in larger, rigorously controlled trials, these findings may have important cost implications for providers, healthcare systems, employers, and policymakers seeking to implement iCBT at scale. Notably, SilverCloud’s multimedia and interactive design, which is likely associated with higher developmental, operational, and end-user costs compared to TWU’s simpler design, did not demonstrate added value in terms of iCBT effectiveness or patient satisfaction. It will be important for future studies to compare the cost-effectiveness of SilverCloud and TWU. In addition, future research using more robust study designs, including the MOST framework and dismantling/additive studies, is needed to understand what components of iCBT maximize adherence and outcomes.

## Funding

The digital cognitive-behavioral therapy clinic is funded by Combined Jewish Philanthropies of Greater Boston (CJP). CJP was not involved in the design or conduct of the current study. This work was supported by Harvard Medical School’s Livingston Fellowship and McLean Hospital’s Pope-Hintz Endowed Fellowship awarded to A.L. Silverman.

## CRediT authorship contribution statement

All authors made substantial contributions to the paper as follows: conceptualization (ALS, JRB, CB), data curation (IF, HB, SM, JRB), formal analysis (ALS), funding acquisition (CB, JB), methodology (ALS, JRB, CB), investigation (IF, HB, SM), project administration (IF, JRB, HB, SM, CB), resources (JRB, CB), supervision (JRB, CB), visualization (ALS), writing-original draft (ALS, IF), writing-review and editing (ALS, IF, JRB, HB, SM, CB). All authors reviewed the results and approved the final version of the manuscript, and agree to be accountable for all aspects of the work in ensuring that questions related to the accuracy or integrity of the work are appropriately investigated and resolved.

## Declaration of Competing Interest

The authors declare the following financial interests/personal relationships which may be considered as potential competing interests: Drs. Courtney Beard and Jacqueline R. Bullis report financial support was provided by Combined Jewish Philanthropies. If there are other authors, they declare that they have no known competing financial interests or personal relationships that could have appeared to influence the work reported in this paper.

## Footnotes

Appendix A Supplementary data associated with this article can be found in the online version at doi:10.1016/j.xjmad.2025.100106.

## Footnotes

1 This exclusion decision was made after conducting power analyses and pre-registering the analytic plan. Thus, power analyses were conducted assuming 106 TWU participants and 91 SilverCloud participants.
