# Supplementary material for: Program evaluation of internet-delivered cognitive behavioral treatments for anxiety and depression in a digital clinic
Source: J Mood Anxiety Disord. 2025 Jan 9;9:100106. doi: 10.1016/j.xjmad.2025.100106 (PMC12244179; doi:10.1016/j.xjmad.2025.100106)
Supplement: Supplementary file 1 — Supplementary material [file mmc1.docx]

Supplemental Materials

**Section S1: Method Notes**

**S1.1 Additional Information on Coaching**

*Training and Supervision*. Training consisted of the following activities: reading articles about internet-delivered cognitive behavioral therapy (iCBT) and reviewing iCBT program content to gain familiarity with CBT concepts, program content, and features; reviewing the digital CBT clinic’s workflow, study protocol, and assessment measures; completing roleplays of relevant study activities (e.g., screening, onboarding, coaching calls, risk assessments) with other coaches, and with the supervising licensed clinical psychologist; and shadowing calls completed by senior coaches. Once training was complete, coaches continued to meet with the licensed clinical psychologist for weekly supervision.

*Coaching Call Procedures*. Coaches were flexible to the patients’ schedules and were able to reschedule most missed calls. Before each call, coaches reviewed the client’s progress over the past week (e.g., number of program logins, modules or activities completed depending on the program). During each call, time was spent reviewing what the patient worked on during the past week and answering questions about the program and CBT skills that were introduced in the past week. Based on this conversation, the coach would then help the patient set goals for the following week regarding which tools they wanted to practice and how much progress they would like to make by the next coaching call. Starting in July of 2021, patients were also given the option to have a one-month follow-up check-in coaching call. This was used by *n*=14 ThisWayUp (TWU) patients (i.e., 13.2% out of the entire TWU sample, and 29.8% of the subset of *n*=47 TWU patients who were offered a one-month follow-up check-in call) and *n*=30 SilverCloud patients (33.7% of the entire SilverCloud sample).

**S1.2 Power Analyses**

*Client Satisfaction and Subjective Engagement*

For analyses of client satisfaction and subjective engagement, power analyses were conducted in G*Power (Version 3.1; Faul et al., 2007) assuming 106 participants in the TWU program and 91 participants in the SilverCloud program. Alpha was set at .05.

For the model testing client satisfaction, we outlined a priori the aim for analyses to be powered to detect a moderate effect size (Cohen’s *d* = 0.5), given that, to our knowledge, there is currently no established clinically important difference for the Client Satisfaction Questionnaire-8 (CSQ-8; Nguyen et al., 1983). Using an independent samples t-test, we have 93.6% power to detect a moderate effect size between the two programs.

For the model testing subjective engagement, we outlined a priori the aim for analyses to be powered to detect a mean difference of at least 0.4 points (10% difference) between the two programs. This difference was selected based on the psychometric evaluation of the Twente Engagement with eHealth Technologies Scale (TWEETS), which compared mean TWEETS scores between participants who adhered to an app (using it at least once per day) and those that did not adhere (Kelders et al., 2020). With a standard deviation of .70, we have 97% power to detect a difference of .4 mean points between the two programs.

*Treatment Effectiveness*

For analyses of treatment effectiveness, power analyses by simulation with 2,000 iterations were run using the mlmpower package in R (Keller, 2024) assuming 106 participants in the TWU program and 91 participants in the SilverCloud program. We outlined a priori the aim to be powered to detect a small effect size (*R^2^* = .02) for the within-between interaction effect of change over time in anxiety, depression, and functional impairment between groups, given that effect sizes for interaction effects tend to be small (Aguinis et al., 2005), and because we expected any between-group differences in outcomes to be small because both iCBT programs have independently demonstrated moderate effects on outcomes of interest. Separately, we established a priori the aim to be powered to detect a medium effect size (*R^2^* = .09) for the within-group effects of change over time in anxiety, depression, and functional impairment, given that prior research has indicated moderate effect sizes for change over time in outcomes for both the SilverCloud and TWU programs.

Intraclass correlations (ICCs) for repeated measures data often reach values of .50 or higher (Arend & Schäfer, 2019). To accommodate uncertainty about this important parameter, the simulations examined ICC values of .50 and .60. The explanatory variables included a within-subject predictor for time during the treatment period (time_TR_) with a random coefficient (except for the model evaluating functional impairment), a within-subjects predictor for time during the follow-up period (time_FU_), a binary level-2 moderator for iCBT group (weighted at 0, with a proportion of .538 to reflect the sample size of the TWU program), and two cross-level interactions for group-by-time_TR_ and group-by-time_FU_ (both weighted at .50). Power analyses indicated that we had at least 80% power to detect small effect sizes for the fixed effects for the within-between interactions, and medium effect sizes for the within-group effects of time_TR_ and time_FU_ (see Table S2).

**S1.3 Auxiliary Variables**

To identify measured variables that may relate to attrition, we assessed whether any demographic variables (i.e., age, gender, sex assigned at birth, sexual orientation, racial-ethnic identity, education, employment, and income) predicted the number of missing assessments. Non-parametric tests were used, given that number of missing assessments was a negatively skewed variable. Education was the only variable found to be significantly related to the number of missing assessments. Specifically, a Kruskal-Wallis rank sum test revealed that patients ranking higher in education ranked significantly lower in their number of missing assessments, *χ*^2^(2) = 11.39, *p* = .003.

With regard to all other demographic variables, Goodman and Kruskal’s gamma tests were not significant for age (*G* = -0.05, 95% CI [-0.16, 0.07]), nor income (*G* = -0.10, 95% CI [-0.24, 0.04]). Wilcoxon rank-sum tests were not significant for gender (*W* = 2,195, *p* = .193), sex assigned at birth (*W* = 2,240.5, *p* = .198), sexual orientation (*W* = 1036, *p* = .3585), nor racial-ethnic identity (*W* = 1,752.5, *p* = .1239); and a Kruskal-Wallis rank-sum test was not significant for employment, *χ*^2^(5) = 4.67, *p* = .458.

**S1.4 Multiple Imputation**

We used the jomo (Quartagno & Carpenter, 2023) and mitml (Grund et al., 2023) packages in R to impute missing scale level scores. For analyses of treatment effectiveness, missing data was imputed using a joint multivariate linear mixed model. In this multiple imputation model, the target variables were the three incomplete Level 1 outcomes (anxiety symptom severity, depression symptom severity, functional impairment) and the incomplete Level 2 auxiliary variable (education). Complete variables by study design were group (TWU vs. SilverCloud), time, as well as the dummy-coded covariate to control for treatment problem(s) targeted by the program (mixed depression and anxiety vs. depression- or anxiety-specific). Group and time were represented with two linear splines in the same model: time_TR_ for the treatment trajectory (coded as 0 for Baseline, integer 1 for mid-treatment (i.e., after one month of treatment), integer 2 for post-treatment (i.e., after two months of treatment), and integer 2 for one-month, two-month, three-month, and four-month follow-ups), and time_FU_ for the follow-up trajectory (coded as 0 for Baseline through post-treatment, and integers 1-4 for one-month through four-month follow-ups). The Level 1 predictors were the fixed effects of group, time_TR_, time_FU_, Group × Time_TR_, and Group × Time_FU_, and random effects for intercept, time_TR_, time_FU_, and the Level 2 predictor was group. We treated target variables as continuous following Grund et al. (2018) to specify the model and impute 20 datasets. Because functional impairment was not assessed at mid-treatment, imputed data at this time point was removed before analyses of all treatment outcomes.

Separately, for analyses of client satisfaction and subjective engagement, we imputed 100 datasets using predictive mean matching. In this imputation model, the target variables were the two incomplete Level 1 outcomes (CSQ-8 total scores and TWEETS mean scores) and the incomplete auxiliary variable (education), and complete variables by study design were group (TWU vs. SilverCloud) and the dummy-coded covariate to control for treatment problem(s) targeted by the intervention (mixed depression and anxiety vs. depression- or anxiety-specific).

**Section S2: Supplemental Results**

**S2.1 Sensitivity Analyses of Between-Condition Effects**

Baseline differences in anxiety, depression, and functional impairment were not significantly different between the two iCBT programs. However, in viewing the plots presented in Figure 2, we observed that the baseline means for anxiety symptoms and functional impairment estimated from the linear spline multilevel models of between-group effects differed by approximately one standard error. We thought that baseline differences of this magnitude might be considered clinically meaningful, even if they were not significant. Thus, in a deviation from our pre-registered analysis plan, we conducted sensitivity analyses of between-group effects of change over time in anxiety symptoms and functional impairment (tested separately) after covarying for baseline differences in anxiety symptoms and functional impairment, respectively. Results remained the same after controlling for these baseline differences (see Table S4).

**2.2 Post-hoc Analyses of TWEETS Subscales**

Independent samples t-tests indicated that SilverCloud (vs. TWU) had significantly greater behavioral engagement (*t*(135)=-2.01, *p*=.046) and cognitive engagement (*t*(135) = -2.13, *p* = .035). However, affective engagement did not differ between iCBT programs (*t*(135) = -1.65, *p* = .102). Further, correlations revealed that client satisfaction was correlated most highly with affective engagement, followed by cognitive engagement, and behavioral engagement (TWU: *r*s = 0.70, 0.64 and 0.56, *p*s < .001; SilverCloud: *r*s = 0.74, 0.62, 0.43, *p*s < .001).

**References**

Aguinis, H., Beaty, J. C., Boik, R. J., & Pierce, C. A. (2005). Effect size and power in assessing moderating effects of categorical variables using multiple regression: A 30-year review. *Journal of Applied Psychology*, *90*, 94-107.

Arend, M. G., & Schäfer, T. (2019). Statistical power in two-level models: A tutorial based on Monte Carlo simulation. *Psychological Methods*, *24*, 1-19.

Faul, F., Erdfelder, E., Lang, A.G., Buchner, A. (2007) G*Power 3: A flexible statistical power analysis program for the social, behavioral, and biomedical sciences. *Behavior Research Methods*, *39*, 175–191.

Grund, S., Lüdtke, O., & Robitzsch, A. (2018). Multiple imputation of missing data for multilevel models: Simulations and recommendations. *Organizational Research Methods*, *21*, 111-149.

Grund, S., Robitzsch, A., & Luedtke, O. (2023). Mitml: Tools for multiple imputation in multilevel modeling [Computer software]. <https://cran.r-project.org/web/packages/mitml/index.html>

Nguyen, T. D., Attkisson, C. C., & Stegner, B. L. (1983). Assessment of patient satisfaction: Development and refinement of a service evaluation questionnaire. *Evaluation and Program Planning*, *6*, 299-313. <https://doi.org/10.1016/0149-7189(83)90010-1>

Kelders, S. M., Kip, H., & Greeff, J. (2020). Psychometric evaluation of the TWente Engagement with Ehealth Technologies Scale (TWEETS): Evaluation study. *Journal of Medical Internet Research*, *22*, e17757. <https://doi.org/10.2196/17757>

Keller, B.T. (2024). Mlmpower: Power analyses and data simulation for multilevel models [Computer software]. https://cran.r-project.org/web/packages/mlmpower/mlmpower.pdf

Quartagno, M. & Carpenter, J. (2023). Jomo: Multilevel joint modelling multiple imputation [Computer software]. https://cran.r-project.org/web/packages/jomo/index.html

**Table S1**. Module Content for THIS WAY UP and SilverCloud Programs

|  |  |  |  |
| --- | --- | --- | --- |
|  | **THIS WAY UP** | | |
| Module | Mixed Depression and Anxiety | Depression-Specific | Anxiety-Specific |
| 1 | Psychoeducation about anxiety and depression, identifying symptoms, fight or flight response, controlled breathing, and physical activity | Psychoeducation about depression, medications, and identifying symptoms of depression | Psychoeducation about GAD, and physical activity/exercise, controlled breathing |
| 2 | Education on the cognitive model, cognitive distortions and introduction to thought monitoring; activity planning | Psychoeducation about low activity in depression, activity monitoring, thought monitoring, education about cognitive distortions, shifting attention, and sleep | Psychoeducation about the fight or flight response, vicious cycle of anxiety, and thought challenging |
| 3 | Thought restructuring | Behavioral activation; Thought restructuring | Thought restructuring |
| 4 | Education about avoidance and safety behaviors, graded exposure, and structured problem solving | Structured problem solving, education about avoidance and facing fears | Education about safety behaviors, graded exposure |
| 5 | Advanced graded exposure | Assertive communication, effective communication skills | Identifying and challenging assumptions and beliefs, acting 'As If', recognizing warning signs of worry |
| 6 | Relapse Prevention | Relapse Prevention | Relapse Prevention |
|  |  |  |  |
| Module | **SilverCloud** | | |
|  | Mixed Depression and Anxiety | Depression-Specific | Anxiety-Specific |
| 1 | Psychoeducation on cycle of anxiety and depression and emotional, cognitive, and behavioral aspects of anxiety, self-monitoring, breathing exercises | Psychoeducation on cycle of depression and emotional, cognitive, and behavioral aspects of anxiety, self-monitoring, breathing exercises | Psychoeducation on cycle of anxiety and emotional, cognitive, and behavioral aspects of anxiety, self-monitoring, breathing exercises |
| 2 | Psychoeducation about anxiety and depression, body scan | Psychoeducation about anxiety and depression, body scan | Psychoeducation about anxiety and depression, body scan |
| 3 | Changing physical sensations, mapping lifestyle choices, progressive muscle relaxation, gradual stimulus control | Changing physical sensations, mapping lifestyle choices, progressive muscle relaxation, gradual stimulus control | Changing physical sensations, mapping lifestyle choices, progressive muscle relaxation, gradual stimulus control |
| 4 | Behavioral activation, mindful eating | Behavioral activation, mindful eating | Graded exposure, psychoeducation on avoidance and safety behaviors |
| 5 | Thought monitoring, meditation exercise | Thought monitoring, meditation exercise | Thought monitoring, meditation exercise |
| 6 | Thought restructuring, mediation exercise | Thought restructuring, mediation exercise | Thought restructuring, mediation exercise |
| 7 | Practical vs hypothetical worries, breathing exercise, self-control desensitization, worry outcome monitoring | Identifying and challenging core beliefs | Practical vs hypothetical worries, breathing exercise, self-control desensitization, worry outcome monitoring |
| 8 | Reflection, goal setting, psychoeducation on importance of social support | Reflection, goal setting, psychoeducation on importance of social support | Reflection, goal setting, psychoeducation on importance of social support |

*Note*. SilverCloud modules could be completed in any order.

**Table S2.** Power Estimates for Simulation Analyses

| Outcome Variable(s) | Fixed Effect | ICC = 0.50 | ICC = 0.60 |
| --- | --- | --- | --- |
| Depression and Anxiety (*M*=6.0, *SD*=4.5) | Time_TR_ | 100% ± 0% | 100% ± 0% |
|  | Time_FU_ | 100% ± 0% | 100% ± 0% |
|  | Time_TR_ × Condition | 81% ± 2% | 87% ± 1% |
|  | Time_FU_ × Condition | 84% ± 2% | 94% ± 1% |
| Functional Impairment (*M*=11.5, *SD*=9.0) | Time_TR_ | 100% ± 0% | 100% ± 0% |
|  | Time2 | 100% ± 0% | 100% ± 0% |
|  | Time_TR_ × Condition | 88% ± 1% | 94% ± 1% |
|  | Time_FU_ × Condition | 99% ± 0% | 100% ± 0% |

*Note*. ICC = Intraclass correlation; M = Mean; SD = Standard deviation;

Time_TR_ = Time during the treatment period; Time_FU_ = Time during the follow-up period.

| **Table S3.** Raw Means and Standard Deviations of Outcomes by Condition Over Time for Intent-to-Treat Sample | | | | | | | |
| --- | --- | --- | --- | --- | --- | --- | --- |
| Outcome | Assessment | THIS WAY UP  (*n*=106) | | | SilverCloud  (*n*=89) | | |
|  |  | *n* | *M* | *SD* | *n* | *M* | *SD* |
| Depression | Pre-treatment (Baseline) | 106 | 8.46 | 4.23 | 87^a^ | 8.64 | 4.70 |
|  | Mid-Treatment (Week 4) | 79 | 6.85 | 4.84 | 71 | 6.23 | 4.52 |
|  | Post-treatment (Week 8) | 77 | 5.07 | 4.24 | 66 | 4.49 | 3.53 |
|  | One-month follow-up | 72 | 5.07 | 3.66 | 56 | 4.25 | 3.31 |
|  | Two-month follow-up | 62 | 4.71 | 3.44 | 46 | 3.80 | 3.46 |
|  | Three-month follow-up | 55 | 4.84 | 4.49 | 39 | 4.67 | 4.21 |
|  | Four-month follow-up | 56 | 5.79 | 4.62 | 35 | 4.71 | 4.94 |
| Anxiety | Pre-treatment (Baseline) | 106 | 9.83 | 4.61 | 87^a^ | 8.81 | 5.16 |
|  | Mid-Treatment (Week 4) | 79 | 7.04 | 4.43 | 68 | 6.02 | 3.89 |
|  | Post-treatment (Week 8) | 77 | 5.38 | 4.01 | 65 | 4.20 | 3.40 |
|  | One-month follow-up | 72 | 5.26 | 3.94 | 56 | 3.80 | 2.93 |
|  | Two-month follow-up | 62 | 5.34 | 3.93 | 46 | 3.87 | 3.20 |
|  | Three-month follow-up | 55 | 5.58 | 4.25 | 39 | 4.51 | 3.69 |
|  | Four-month follow-up | 56 | 5.71 | 3.95 | 36 | 5.00 | 5.28 |
| Functional Impairment | Pre-treatment (Baseline) | 106 | 16.08 | 9.32 | 87^a^ | 14.23 | 9.09 |
|  | Post-treatment (Week 8) | 77 | 11.66 | 7.77 | 65 | 9.17 | 8.41 |
|  | One-month follow-up | 71 | 10.94 | 8.48 | 56 | 8.34 | 7.46 |
|  | Two-month follow-up | 62 | 10.40 | 8.59 | 46 | 7.11 | 5.94 |
|  | Three-month follow-up | 55 | 9.56 | 7.52 | 39 | 8.77 | 8.37 |
|  | Four-month follow-up | 56 | 10.34 | 7.68 | 36 | 8.67 | 8.54 |
| Subjective Engagement | Post-treatment (Week 8) | 73 | 2.56 | 0.77 | 64 | 2.80 | 0.58 |
| Satisfaction | Post-treatment (Week 8) | 73 | 25.83 | 4.10 | 64 | 26.95 | 4.29 |

*Note*. ^a^ Two participants did not answer all depression, anxiety, and functional impairment items at baseline.

| **Table S4**. Sensitivity Analyses of Linear Spline Multilevel Models for Fixed Group × Time Interaction Effects | | | | | | | | |
| --- | --- | --- | --- | --- | --- | --- | --- | --- |
| Outcome | Phase | Effect | B (SE) | 95% CI | | *p* | GMA *d* | |
| Anxiety | Treatment | Group × Time | 0.04 (0.22) | [-0.58, 0.65] | .911 | | | 0.02 |
|  | Follow-Up | Group × Time | -0.17 (0.22) | [-0.61, 0.27] | .441 | | | -0.13 |
| Functional Impairment | Treatment | Group × Time | -0.17 (0.57) | [-1.29, 0.96] | .769 | | | -0.04 |
|  | Follow-Up | Group × Time | 0.04 (0.36) | [-0.68, 0.77] | .904 | | | -0.02 |

*Not*e. GMA = Growth-modeling analysis
